# The role of geography, environment, and genetic divergence on the distribution of pikas in the Himalaya

**DOI:** 10.1002/ece3.6007

**Published:** 2020-01-22

**Authors:** Nishma Dahal, Sunil Kumar, Barry R. Noon, Rajat Nayak, Rinzin Phunjok Lama, Uma Ramakrishnan

**Affiliations:** ^1^ National Centre for Biological Sciences TIFR, GKVK campus Bangalore India; ^2^ Nature Conservation Foundation Mysore India; ^3^ Manipal Academy of Higher Education Manipal India; ^4^ Natural Resource Ecology Laboratory Colorado State University Fort Collins CO USA; ^5^ Foundation for Ecological Research, Advocacy and Learning Morattandi Tamil Nadu India; ^6^ Institute of Forestry Pokhara Campus Pokhara Nepal

**Keywords:** Indicator species, Kernel density, niche breadth, noninvasive, pika

## Abstract

Pikas (*Ochotona* Link, 1795) are high‐altitude specialist species making them a useful bioindicator species to warming in high‐altitude ecosystem. The Himalayan Mountains are an important part of their range, supporting approximately 23%–25% of total pika species worldwide, yet we lack basic information on the distribution patterns. We combine field‐based surveys with genetics‐based identification and phylogeny to identify differences in species‐environment relationships. Further, we suggest putative evolutionary causes for the observed niche patterns.

**Location:**

Himalayan high‐altitude region.

**Methods:**

We sampled 11 altitudinal transects (ranging from ~2,000 to 5,000 m) in the Himalaya to establish occurrence records. We collected 223 species records using genetic analyses to confirm species' identity (based on some invasive and mostly noninvasive biological samples). Niche and geographic overlap were estimated using kernel density estimates.

**Results:**

Most pikas in the Himalaya span wide elevation ranges and exhibit extensive spatial overlap with other species. However, even in areas of high species diversity, we found species to have a distinct environmental niche. Despite apparent overlapping distributions at broad spatial scales, in our field surveys, we encountered few cases of co‐occurrence of species in the sampled transects. Deeply diverged sister‐species pair had the least environmental niche overlap despite having the highest geographic range overlap. In contrast, sister‐species pair with shallow genetic divergence had a higher environmental niche overlap but was geographically isolated. We hypothesize that the extent of environmental niche divergence in pikas is a function of divergence time within the species complex. We assessed vulnerability of species to future climate change using environmental niche and geographic breadth sizes as a proxies. Our findings suggest that *O. sikimaria* may be the most vulnerable species. *Ochotona roylii* appears to have the most unique environmental niche space, with least niche overlap with other pika species from the study area.

## Graphical abstract

Pikas (Ochotona Link, 1795) are high‐altitude specialist species making them a useful bioindicator species to warming in high altitude ecosystem. The Himalayan Mountains support approximately 23%–25% of total pika species worldwide, yet we lack basic information on the distribution patterns. The study addresses this information gap and identifies patterns of pika species distribution and provides an evolutionary perspective to the identified pattern.

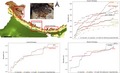

## 1 INTRODUCTION

A long‐standing interest in ecology is the question of what sets geographic limits of a species range (Darwin, 1859). At an intermediate temporal scale (multiple generations), a species range can be highly labile, often varying with changing environmental conditions (Brown, Stevens, & Kaufman, 1996; Zacaï et al., 2017). At a spatial scale, species range is fixed at upper and lower elevation limits by a complex interplay of abiotic and biotic factors (Ettinger & Hillerislambers, 2017; Jankowski, Robinson, & Levey, 2010). A common approach to study impacts of climate change uses climate as a sole determinant (Morán‐Ordóñez, Briscoe, & Wintle, 2018) which probably is overly simplified prediction (Ettinger & Hillerislambers, 2017). Deconstructing the dynamics of species distribution patterns is, therefore, particularly important in regions with rapidly changing environment. Mountainous landscape represents a heterogeneous environment where plant and animal community composition can change abruptly with changes in elevation. Many extensive mountain ranges are regions of high conservation priority (Körner, 2004) as they host high species diversity and are under threat of rapid climate change. The upper elevation zones of mountains are particularly vulnerable to climate change as they are experiencing more rapid rates of warming (elevation‐dependent warming; Pepin, Lundquist, 2008; Thompson et al., 1999; Thuiller, Lavorel, Araújo, Sykes, & Prentice, 2005). Climate change may result in shifts in the distribution and abundance of species, thereby altering community composition (Chen et al., 2009; Moritz et al., 2008; Parmesan & Yohe, 2003). However, our understanding of the existing distribution patterns of plant and animal communities from the Himalayan Mountains is still preliminary because of limited studies (Anthelme & Lavergne, 2018). We studied the distribution of high‐altitude mammalian specialist species in climatically unstable Himalayan mountaintops to understand the processes that might have led to the present distribution pattern of these species.

Spatial variation in environmental conditions is driven by topographic variation (Graae et al., 2018) such as elevation change which is often identified as key correlates of the distribution of plant and animal diversity in mountains (e.g., SÁnchez‐Cordero, 2001). The distribution of biota and community composition in the heterogeneous mountainous landscape is influenced by the complex interplay of abiotic and biotic factors (Aiello‐Lammens et al., 2017). Abiotic factors include environmental drivers associated with topography and elevation, while biotic factors are represented by mutualistic and competitive interactions with co‐occurring species, for example, predation, host‐parasite interactions, and facilitation (reviewed in Wisz et al., 2013). The role of evolutionary history and biogeography in determining regional diversity has been well documented (Ricklefs & Schluter, 1993; Webb, Ackerly, McPeek, & Donoghue, 2002). The most direct explanation for the changes in species diversity along elevational gradients is changes in environmental factors related to physiological constraints (Jankowski et al., 2010) or habitat specialization (Presley, Cisneros, Patterson, & Willig, 2012). Species diversity patterns vary across the spatiotemporal scale. Scale‐dependent variation in estimated regional diversity (gamma diversity) may be apparent even in a single study site (alpha diversity) (Powell, Chase, & Knight, 2013) and may affect conservation management decisions (Socolar, Gilroy, Kunin, & Edwards, 2016). It is important to understand the spatiotemporal setting which ultimately drives the ecological processes and evolution of a species. Geographic data on distribution are vital for any ecological studies, but it is poorly documented for most high‐altitude specialist species.

An integrative approach to understand the evolutionary processes that shaped the current distribution of animal diversity in montane regions is needed which includes distribution, ecological, and historical perspectives. A species' elevational distribution, niche breadth, and niche relationships provide information on the current responses to both biotic and abiotic environmental drivers. Studies of their evolutionary relationships provide insights into the historical processes that have contributed to the current niche relationships among sympatric species. From an evolutionary perspective, the composition and diversity of a species complex at a local scale are influenced by selection, drift, speciation, and dispersal (Vellend, 2010). Species whose distributions are limited to mountaintops are expected to have a narrow fundamental niche given their narrow physiological range. As a result, they may be particularly vulnerable to both ecological and evolutionary changes. We studied the distribution of high‐altitude mammalian specialist species in Himalayan mountaintops to understand the process that might have shaped the present distribution pattern of these species. In our research, we integrate ecological and evolutionary information to understand the distribution pattern and degree of niche partitioning in a species complex consisting of six high‐altitude pikas species in the Indian and Nepalese Himalayan Mountains.

Species in the genus *Ochotona*, commonly known as pikas (Order Lagomorpha, Family Ochotonidae), are high elevation specialists found in the plateau steppe and talus of the Holarctic region (Lissovsky, 2016). Studies in North America have documented their vulnerability to global warming (Beever, Ray, Mote, & Wilkening, 2010; Beever, Ray, Wilkening, Brussard, & Mote, 2011). The adaptive capacities of pikas to climate change are of particular concern in Asia since this region includes more than 90% of global pika species (Lissovsky, 2016). In particular, the Himalaya and neighboring mountain ranges in Asia are an important part of pikas' distributional range, as they support approximately 25% of the total pika species (Lissovsky, 2016). This raises the interesting ecological question about how such similar species achieve coexistence, how the species share space and resources, the relative contributions of niche differentiation versus competitive exclusion, and the species' vulnerabilities to rapid environmental change.

Pikas species in the Himalayan region belong to two subgenera—*Ochotona* and *Conothoa—*with co‐occurring species in the two subgenera coexisting presumably by broad differences in their ecological niches. Species belonging to *Ochotona* subgenera primarily occur in meadow habitats whereas those belonging to *Conothoa* subgenera occur mostly in talus patches (Lissovsky, 2016). Thus, local coexistence among pikas of divergent lineages facilitated by differences in habitat use may allow overlapping geographic ranges. In addition, the hypothesis of phylogenetic niche conservatism predicts higher niche overlap among congeners because of a shared evolutionary history (Losos, 2008). In this study, we combined information from field‐based surveys with phylogenetic analyses to identify differences in species' distribution due to environmental factors and to identify putative evolutionary causes for the identified relationships between species' ecological niches. Specifically, we estimated the distribution of pikas along environmental gradients of the Himalaya and asked (i) if different species of pikas occupy distinct environmental niche; (ii) if congeners show higher environmental niche overlap compared with more distantly related species; and (iii) if coexisting species (with high geographic overlap) show higher niche overlap.

## 2 METHODS

### 2.1 Sampling methods

Two methods were applied to assign species identification to pika‐inhabited locations. We used noninvasive fecal sample as well as live‐trapping and tissue collection to identify species at pika‐inhabited locations. Locations near the transects where active pika presence was perceived based on fecal pellet abundance and sighting frequencies (seven sites in Eastern Himalaya, five sites in Central Himalaya, and seven sites in Western Himalaya) were selected for trapping. Trapping was done for a minimum of three days at each location. Trapped individuals were released after taking body measurements and an ear punch. Tissue samples were collected in 95%–100% ethanol, and fecal samples were collected in silica gel. The samples were stored at −20°C after reaching the laboratory. Fecal pellets initially stored in ethanol gave low DNA yield. Consequently, the sites sampled in the first year (2010) from Kyongnosla Alpine Sanctuary and surroundings in East Sikkim were resampled in 2013 and stored in silica gel. DNA was isolated from 440 fecal pellet samples and 59 tissue samples collected during field surveys over four years, from 2010 to 2014 (Supplementary Tables 1 and 2). Tissue samples were used to generate longer sequence lengths for confirmation of species identity from those locations.

**Figure 1:**
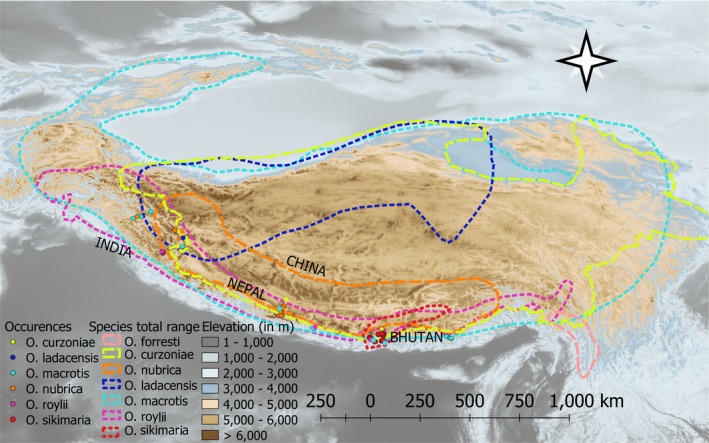
The map shows sampled locations plotted on near 30 m resolution elevation data (source: Shuttle Radar Topography Mission, SRTM). Points and polygons are color‐coded to show species‐wise occurrences and their overall range (source: IUCN and Dahal et al., 2017). The map was made in QGIS version 3.0.0‐Girona (URL: http://qgis.org)

**Figure 2:**
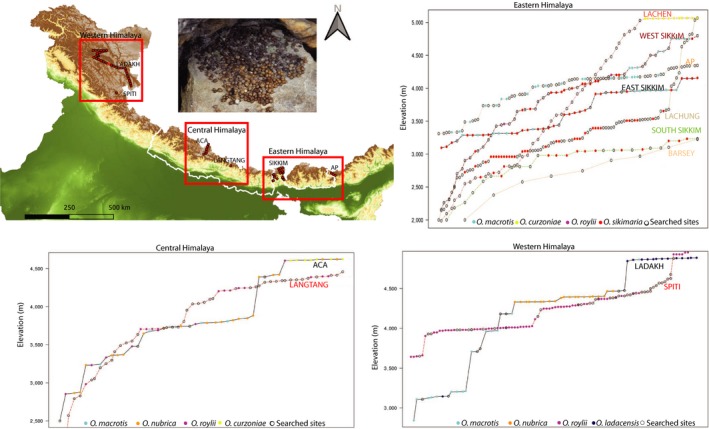
The map shows sampled locations plotted on near 30 m resolution elevation data (source: Shuttle Radar Topography Mission, SRTM) in Eastern, Central, and Western Himalaya. The photograph on the top left shows the pellet pile captured in field. Different transects in the three transect plots (Eastern, Central, and Western Himalaya) are color‐coded, and each transect shows searched sites and sampled species in different colors

The strategy to collect fecal pellets was designed to sample the maximum accessible altitudinal range from each of the eleven sites. Altitudinal transects were walked starting from 2,000 m to 5,000 m in most regions, depending on accessibility and maximum elevation of the region. Sampling was carried out primarily by walking trekking trails. In locations where we did not achieve much elevation gain following trekking trails, sampling was carried out using the road. Sikkim Himalaya was searched more intensely since this region was believed to have the highest pika species richness; five species have been reported from this region (Figures 1 and 2; sampling details in Supplementary Table 1). In the Western Himalaya, less steep mountain gradients resulted in sampling a more restricted elevation range over large distances. Therefore, continuous altitudinal transects could not be laid, and sampling had to be carried out by accessing many areas by road. Lower elevation regions, approximately between 2,000 to 3,000 m, were poorly sampled, as areas below 3,000 m, near roads were mostly settlements or cliffs. We acknowledge the sampling limitations in the lower elevations of Arunachal Pradesh (Eastern Himalaya) and Ladakh and Spiti (Western Himalaya). Future studies on the distribution of pikas should focus more intensely on these regions to fill the gap. Based on our knowledge on pikas from museum collection records from this region at Bombay Natural History Society and Zoological Survey of India, we do not believe our sampling failed to detect any previously described species.

### 2.2 DNA isolation, amplification, sequencing, and species identification

Mitochondrial DNA was targeted for species identification for two reasons; 1) the cytochrome *b* (cyt *b*) gene sequence is available for pika species; and 2) mitochondrial DNA is more abundant than nuclear DNA, making it an ideal marker when the source is degraded fecal samples. Extraction was done using a commercially available DNA stool and tissue kit (Qiagen Inc.), following the manufacturer's instructions. Pikas defecate in "latrines," and each pile contains between 4 and >100 pellets. We collected pellets from 440 distinct latrine piles. From each pile (Figure 2), 5 to 15 pellets, fresh‐appearing pellets were collected in a single collection tube, and 4 to 5 fecal pellets were used for DNA extraction per reaction. Out of the 440 fecal samples (from distinct latrines) processed for DNA extraction, we successfully generated sequences from 312 samples. The success rate appeared to be dependent on sampling location and time of extraction. DNA extraction from samples immediately after field collection gave a better yield. Samples from Western Himalaya showed higher success, relative to wetter regions of Eastern Himalaya (Supplementary Table 2). We also included occurrences from 59 tissue samples.

We used a vertebrate generic forward primer (L14724) and pika‐specific reverse primer (designed by Lissovsky, Zoological Museum of Moscow State University, unpublished) to amplify 425 bp of cytochrome *b* (cyt *b*) from the pellet samples (primers details in Supplementary Table 2). We were successful in amplifying fragments ranging from 230 to 513 bp. Vertebrate generic primers (L14724/H15915, and internal primers L15513 and H15149) (Irwin, Kocher, & Wilson, 1991) were used to amplify cyt *b* gene sequences from tissue samples (following protocol explained in Dahal et al., 2017). To avoid nonspecific amplification, touchdown PCR was optimized for fecal pellets with starting annealing temperature of 65°C, which was lowered at every cycle until it reached an annealing temperature of 55°C (modified from Murphy & O'Brien, 2007). PCR products were visualized on a 2% agarose gel and sequenced on an automated sequencer 3730 Genetic Analyzer (Applied Biosystems, Thermo Scientific, USA) in both directions. The sequences were aligned using de novo assembly in Geneious 7.1.9 (http://www.geneious.com/) after checking for insertion, deletion, and stop codons.

**Figure 3:**
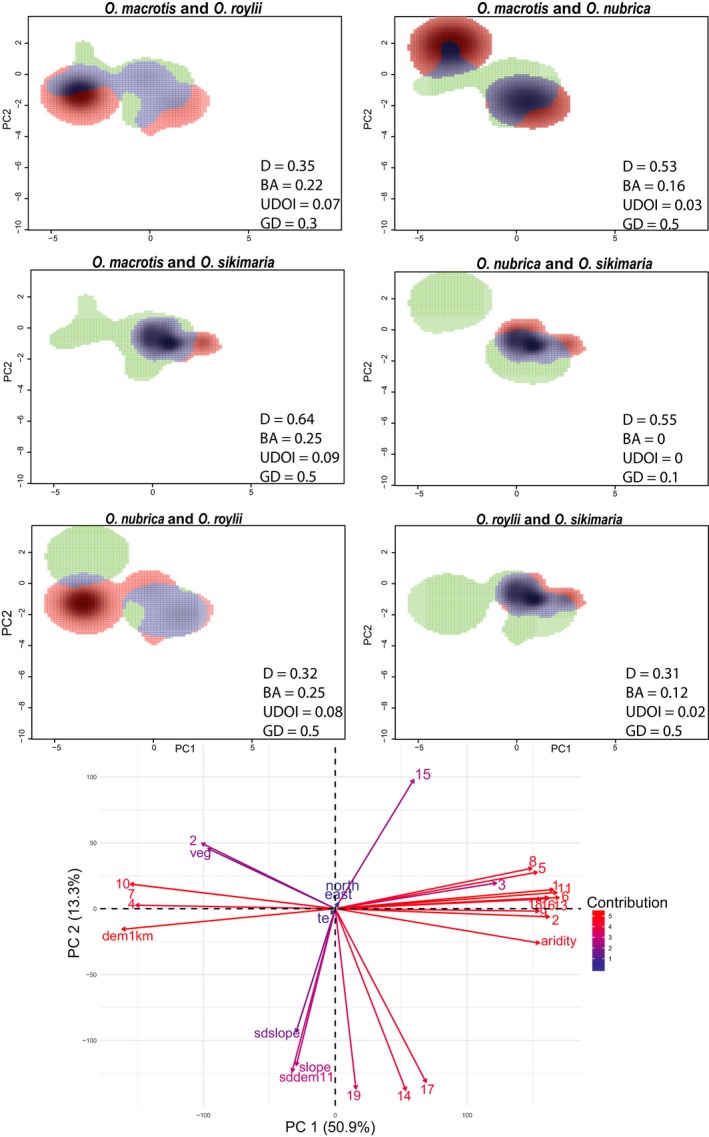
Niche overlap of different pika species in the environmental space of the study area (PCA—env). The green color depicts the niche space of the first species, red of the second species and the overlapping range is shown in blue. Niche overlap (D) values are presented for comparisons of similarity of species 1 and 2. Paired overlap values which significantly (*p* < 0.05) differ from the associated null distribution. The significance test was done for both directions of species pairs (species 1 to 2 and species 2 to 1), and all comparisons were significant in both directions. BA and UDOI correspond to two indices of geographic overlap. Average pairwise genetic distances (GD) are the number of nucleotide differences in cytochrome *b* gene sequences. The correlation circle in the bottom shows variables contributing to the PC axes of the climatic niche of the study area. The arrow depicts the direction of correlation (same direction indicates a high correlation). Red to blue color (high to low) indicates variables contributing to the axes (for more clarity refer Supplementary Figure 2). All the bioclimatic variables are coded as numbers

The sequences were aligned and compared with existing cytochrome *b* sequences of pikas and hares available in GenBank. Accession numbers of the sequences are provided in the Supplementary Figure 1a–f. A rapid bootstrap maximum likelihood (ML) tree was built in RAxML GUI v1.3 (Silvestro & Michalak, 2012). The position of the sample in the tree was ascertained using a resampling test (bootstrap) where support value >70 (out of 1,000 iterations, 70% of the time the same node was recovered) were considered to assign species, following usual practice (Chen et al., 2018). Considering the taxonomic anomalies associated with this group, assigning species was complicated for some taxa. Thus, we established a criterion for taxonomically ambiguous groups. Taxonomic identity of *O. himalayana* is debatable; we followed the most recent classification by Lissovsky (2014) and grouped it with *O. roylii*. In another case, *O. nubrica* and *O. curzoniae* represent a potential case of hybridization—they are morphologically distinct but have similar mitochondrial DNA (Lissovsky, Yatsentyuk, & Koju, 2019). Therefore, along with genetic species identification, we also used information from direct observations to classify these two species. We removed occurrence points for these two species at locations where we had no direct sightings. The placement of samples in the tree is presented by transect in Supplementary Figure 1a–f. Quantification of genetic relatedness of species relationship was made using estimates of average pairwise genetic distance (presented in Figure 3). Pairwise genetic distances were calculated from downloaded cytochrome *b* sequences from GenBank. The sequences were aligned using ClustalW algorithm, and pairwise p distance was calculated in MEGA7 (Kumar, Stecher, & Tamura, 2016).

### 2.3 Environmental data

We collected 27 climatic, topographic, and remotely sensed variables' geospatial data including 19 bioclimatic variables, vegetation cover, and various topographic variables (details in Supplementary Table 4) (Fick and Hijmans, 2017; SRTM, 2017; Trabucco and Zomer, 2009; Friedl and Sulla‐Menashe, 2018). The elevation at sampled locations was recorded in the field with a handheld geographic positioning system (Garmin GPS, 60Cx, Figures 1 and 2). The difference between the maximum and minimum elevation recorded for the species was used to define species' elevational range for the study area.

Covariates representative of potential niche dimensions were estimated from raster layers of approximately 1 km spatial resolution (details in Supplementary Table 4). The total number of occurrences per species across all 1 km grid cells of the study area in which a species was detected is given in Table 1. Occurrence records of species from less than two localities in the sampled transects (*O. ladacensis* and *O. curzoniae*) were excluded for niche analyses as these would capture a limited environmental range of species. Moreover, the study area represented a small part of the range edge of the species that were excluded from the niche analyses (Figure 1). This left us with four species for which niche analyses and comparisons were made.

**Table 1:** Number of occurrences per pika species

Species	Occurrences per species	Number of occurrences after spatial filtering
*O. curzoniae*	11	3
*O. ladacensis*	4	2
*O. nubrica*	31	8
*O. sikimaria*	64	34
*O. roylii*	56	25
*O. macrotis*	57	27

### 2.4 Niche overlap and breadth

A species' niche space and niche overlap with other species was quantified using an ordination method used to reduce the dimensionality of environmental variables as described by Broennimann et al. (2012). The method involved estimating the smoothened occurrence density (Z_ij_ index) of each species, subsequently plotted on the gridded niche space. We used the first two axes of the centered principal components to integrate 27 environmental variables (Supplementary Table 4) and define the niche space of the study area. Niche overlap of each species pair was estimated using Schoener's D metric (Schoener, 1970; Warren, Glor, & Turelli, 2008). Schoener's D metric varies between 0 (dissimilar niche) and 1 (identical niche). Schoener's D is easy to interpret and has a long history of use compared with other matrices. Niche quantification and comparison, analyses of contribution of variables to PCA‐env (Figure 3 and Supplementary Figure 3), statistical test of similarity, and visualization of niche overlap (Figure 3) were performed using ecospat package version 3.0 (Broennimann, Valeria, & Guisan, 2018). Correlation plot of contributing variables to PC axes (presented in Figure 3) was made using package factoextra v1.0.5 (Kassambara & Mundt, 2017). All analyses were performed using R 3.3.0 (R core team, 2016) using RStudio Version 1.0.136 (RStudio Team, 2016). A statistical test of niche similarity (described in Broennimann et al., 2012) was performed to determine if the observed D was more similar than expected by chance, accounting for the available surrounding niche space. The observed D was compared with simulated D achieved by shifting the entire occurrence density of one species randomly 1,000 times. A significant (*p* < 0.05) difference in observed and simulated D would indicate the niches are more similar or different than expected by chance. Finally, niche breadth (NB) was calculated as a variance of PC 1 scores for each species (adapted from Gómez, Tenorio, Montoya, & Cadena, 2016).

### 2.5 Geographic range and overlap

We calculated kernel‐based home‐range estimates for high use areas (defined by 95% probability contours) for each species using spatial information collected through GPS (Fieberg & Kochanny, 2005). These home‐range values were compared with niche breadth to explore the relationship between niche breadth and geographic range of a species. To calculate the geographic overlap between species pairs, we first estimated space utilization distance (UD̂) between high use areas (within 95% probability contours) using Kernel density estimation. These UD̂s were then used to calculate geographic overlap. We used two UD‐based indices (Bhattacharya Affinity—BA and Utilization Distance Overlap Index—UDOI) (Bhattacharyya, 1943; Hurlbert, 1978) and an index (PHR) (Ostfeld, 1986) that describes the relative probability of space‐use between species pairs to estimate geographic space overlap. BA and UDOI are nondirectional and range between 0 and 1 (where 0 indicate no overlap, and 1 indicates complete overlap). The PHR index is directional and has two values per species pair—ranging between 0 and 1. PHR1,2 indicates probability of species 2 being located in spices 1's range, and vice‐versa. These three indices are discussed and recommended by Fieberg and Kochanny (2005). These indices are used in the estimation of home‐range overlap for GPS data. We used cylindrical equal area projection for all the spatial data and analyzed using R statistical software.

## 3 RESULTS

### 3.1 Distribution along the altitudinal gradient of the Himalaya

We captured six species of pikas in the sampled transects (Figures 1 and 2; Supplementary Table 1). The sampled location includes a small part of the total range of *O. curzoniae*, *O. ladacensis,* and *O. macrotis* (Figure 1). Therefore, we removed *O. curzoniae* and *O. ladacensis*, with occurrence record of three and two for niche analyses. We included *O. macrotis* in the niche analyses as it had a larger sample size, collected from across the known elevation range of the species (Supplementary Figure 2). We did not find pikas in Barsey Rhododendron Sanctuary (West Sikkim) despite having an altitudinal range of 2,500 to 3,200 m, similar to other active pika habitats. A total of 223 occurrences were compiled which after spatial filtering resulted in 99 occurrences (Table 1). Phylogenetic positions of the samples collected from transects with more than two species are presented in supplementary Figure 1a–f.

Four out of six species, *O. sikimaria*, *O. roylii, O. nubrica,* and *O. macrotis*, had wide altitudinal ranges varying from 2,500 to 4,700 m, 2,800 to 4,900 m, 2,800 to 4,600 m, and 3,000 to 4,800 m, respectively (Supplementary Figure 2). These four species have an overall elevation range of approximately 1,800 m (Table 2). Their abundance (based on pellet encounter rate) peaked at the midelevation within their elevation range (Supplementary Figure 2). The other two species, *O. curzoniae* and *O. ladacensis,* had narrower elevational ranges and were recorded from higher elevations of the trans‐Himalayan region in Indian and Nepalese Himalayan range.

**Table 2:** Estimated values of niche breadth (NB) and altitudinal ranges of four Himalayan pika species

Species	Elevational breadth (m)	Niche breadth	Geographic breadth
*O. roylii*	1,762	3.90	0.35
*O. macrotis*	1,747	2.38	0.34
*O. nubrica*	1,765	4.55	0.30
*O. sikimaria*	2,110	0.91	0.01

Note

Elevational breadth was calculated as difference of maximum and minimum elevation at which species occurrence was detected and niche breadth as variance of PC1. Geographic range size (breadth) was calculated using kernel density estimates scaled by total range size.

### 3.2 Niche properties

The niche space of the study area was defined by the first two axes of the principal components which explained 64.2% (PC1—50.9% and PC2—13.3%) of environmental variance of the study area through orthogonal and linear combinations of the original environmental variables (Figure 3). Environmental variables characterizing the first principal component (PC 1) included temperature and precipitation related variables and change in elevation (Supplementary Figure 3). The second PCA axis explained approximately 13.1% of the variation in covariate space and was most strongly related to precipitation change and topographic variables. In general, this axis represents topographic heterogeneity.

### 3.3 Niche and geographic breadth and overlap

Niche breadth is often correlated with geographic range size (Slatyer, Hirst, & Sexton, 2013; Thompson, Gaston, & Band, 1999). *Ochotona sikimaria* has least geographic breadth, and as expected, the estimated niche breadth was minimum for *O. sikimaria* despite having a broad elevational range (Table 2). However, estimated niche breadth showed high variability among species with larger geographic breadth. *Ochotona nubrica* had comparatively smaller geographic breadth than the other two species, but surprisingly *O. nubrica* showed highest niche breadth.

Niche overlap (D) patterns among pika species from the Himalaya and surrounding ranges are presented in Figure 3 along with two estimates of geographic overlap and genetic distance. The highest niche overlap of D = 0.64 was observed between *O. sikimaria* and *O. macrotis. Ochotona macrotis* showed highest overlap with all other species under comparison whereas *O. roylii*, a congener of *O. macrotis,* showed least overlap with all other species. Comparisons of niche overlap values among two pair of congeneric species, *O. roylii* and *O. macrotis* and, *O. sikimaria* and *O. nubrica,* showed contrasting result with higher overlap for the first pair with higher genetic distance compared with the latter conger pair with low genetic distance. Based on all pairwise comparison of environmental niche overlap of pikas found in the study area, we rejected the null hypothesis of niche similarity between pika species from the study area (significance tested at *p* < 0.05) (Figure 3).

Two nondirectional indices (BA and UDOI) and one directional index (PHR) were calculated to estimate the geographic overlap between species pairs. All the indices estimated no geographic overlap between *O. sikimaria* and *O. nubrica*, followed by the minimal overlap between *O. roylii* and *O. sikimaria* (Supplementary Table 5 and Figure 3). The highest geographic overlap was estimated for the following pairs—*O. macrotis/O. sikimaria,* followed by *O. nubrica/O.roylii* and *O. macrotis/O. roylii* using both nondirectional indices. Directional index (PHR) estimated the highest percentage of *O. roylii* (PHR = 1) and *O. macrotis*' (PHR = 0.97) geographic range used by *O. sikimaria. Ochotona roylii* was also estimated to use (PHR = 0.5) the geographic range of *O. nubrica* (Supplementary Table 5).

## 4 DISCUSSION

The distribution and diversity of animal taxa occupying extensive elevational gradients reflect the interacting influence of multiple environmental factors (Jankowski, Londoño, Robinson, & Chappell, 2013). Species segregate along the environmental gradients of the mountains initiating parapatry. Isolation in the mountains provides opportunities for allopatric diversification. Additionally, the distribution of species is influenced by the regional species pool and the phylogenetic distinctiveness among the community members. Communities may consist of closely related species if environmental filtering shapes their distributions (Warren, Cardillo, Rosauer, & Bolnick, 2014). In contrast, coexisting species are expected to be less related (phylogenetic overdispersion) if competition predominates (Harrison & Cornell, 2008). We combined information on the geographic range, niche similarity, and evolutionary relationship of Himalayan pikas to understand the distribution patterns.

### 4.1 Distribution along the environmental gradient of study area

The lowest elevation at which we first recorded the presence of pikas in the Himalaya was 2,600 m (Figure 2 and Supplementary Figure 2). The lower range limit of pikas in the Western Himalaya, however, needs additional confirmation. We confirmed the occurrence of six species of pikas although IUCN lists seven species of pikas from the study area (IUCN, 2017). Our study and museum collections from this region failed to detect *O. forresti*. In fact, recent study on taxonomic assessment of sample (identified as *O. forresti*) in Smithsonian Institution's National Museum of Natural History collected from Eastern Himalaya (surrounding AP transect—refer to Figure 2) is identified as a new subspecies, *O. macrotis gomchee* (Lissovsky et al., 2017). Therefore, the occurrence of *O. forresti* from the study area is likely to be mistaken.

Our study confirmed wide elevation range of four pika species in comparison with elevation range mentioned by IUCN (IUCN, 2017). Elevation range width of these four species was between 1,700 and 2,100 m (Table 2). The elevation range of *O. nubrica* appears to be wider than previously known range. The two trans‐Himalayan species, *O. curzoniae* and *O. ladacensis*, were found only above 4,500 m elevation in the study area, while IUCN elevation range appears to be much wider (Supplementary Figure 2). Our survey efforts included a small part of the range edge of *O. ladacensis* and had a small sample size for this species, so we do not have a reliable estimate of overall elevational distribution. Many low altitude trees, reptiles, and birds in the Himalaya occupy very narrow elevation ranges (Acharya, Chettri, & Vijayan, 2011; Acharya, Sanders, Vijayan, & Chettri, 2011; Chettri, Bhupathy, & Acharya, 2010). Compared with the elevational ranges of other species from the region, pikas appear to have a large elevation range. The comparatively large elevational range sizes of pikas may be related to Rapport's rule—with increasing elevation, breadth of climatic conditions experienced by a species is expected to be higher (Stevens, 1992).

The elevational distribution of pikas occurrences collectively suggests the absence of spatial segregation between the two species with wide ranges*.* In contrast, Kawamichi (1971), who sampled a continuous elevation transect, detected elevation segregation of these two sister species, *O. roylii* and *O. macrotis*. In West Sikkim, where we sampled a continuous elevational transect, we found fecal pellets of *O. roylii* at lower elevations and *O. macrotis* pellets at higher elevations. However, from the pellet encounter rate in the continuously laid West Sikkim transect in Eastern Himalaya, the density of *O. roylii* (2 pellets out of 21 pellets successfully identified, Figure 2—West Sikkim transect) appears to be much lower than encounter rate of *O. macrotis*. Therefore, our survey results suggest that *O. roylii* and *O. macrotis* exclude each other in regions where species' distributions could potentially overlap. The result of the overall transects suggests environmental niche overlap to be minimal for the two species.

### 4.2 Role of evolutionary relationship, niche similarity, and geographic overlap on the distribution pattern

Pikas found in the study area belong to two subgenera and have known subgenera‐specific ecological differences. Species within the subgenus *Conothoa* occupy rock piles (excluding *O. ladacensis*), and species within subgenus *Ochotona* occur largely in meadows (Lissovsky, 2016). We expect that differences in habitat use between species of different subgenera might result in distantly related species having least niche similarity. However, species‐specific niche overlap patterns seem to be more apparent. *Ochotona roylii* showed least niche overlap, and *O. macrotis* showed highest overlap with all species under comparison, irrespective of genetic relationship.

Niche overlap values ranged between 0.25 and 0.64 (0—completely dissimilar niche and 1—identical niches), indicating that pika species in the Himalaya occupy distinct environmental niche spaces. Similarly, estimates of geographic overlap ranged between 0 and 0.25 (considering nondirectional estimates of BA and UDOI, where 0 indicate allopatric range and 1 indicates complete geographic overlap), indicating small local scale sympatry between species (Figure 3). Throughout our study, we did not encounter species coexistence locally, except in one location in West Sikkim where we sampled two piles of *O. sikimaria* (a meadow dweller) pellets in *O. macrotis*' habitat (talus patch). The patterns of niche divergence and similarity coupled with the geographic distribution of the sister‐species pairs provide important insights into their niche evolution and mode of speciation. Our results suggest contrasting evidence of phylogenetic clustering and overdispersion of environmental niches among the congeners in two subgenera. Deeply diverged (with genetic distance of 0.3) sister species, *O. roylii* and *O. macrotis*, indicate overdispersion, whereas recently diverged sister species (with genetic distance of 0.1), *O. sikimaria* and *O. nubrica*, indicate phylogenetic clustering of their environmental niches.

*Ochotona sikimaria* and *O. nubrica* represent the most recent species in the family Ochotonidae with a time of divergence estimated as 1.3 myr (0.8–1.7 myr) (Dahal et al., 2017)*.* Phylogenetic clustering in recently diverged species has been observed in herbaceous plants such as Banksias (Merwin, He, & Lamont, 2012). In contrast to Banksias, where the recently diverged species exhibited local coexistence, the distributions of *O. nubrica* and *O. sikimaria* are geographically distinct (geographic overlap value of 0; Figure 1 and 3). Niche breadth was highest for *O. nubrica* which is noticeable even in the niche comparison plots (Figure 3). The two sampled localities (in the Central Himalaya and Western Himalaya) appear to be having distinct niche spaces, which perhaps has contributed to broader niche breadth. Further, niche comparison analyses of *O. nubrica* shows highest overlap with congener, *O. sikimaria*. But, the niche overlap appears to be only at one distinct patch (Figure 3)—*O. sikimaria* shares higher niche similarity with niche space of *O. nubrica* sampled from Central Himalaya. We had split the occurrences of *O. nubrica* from the Western Himalaya and Central Himalaya to investigate which region contributes to niche similarity with *O. sikimaria*. Overall, *O. nubrica* and *O. sikimaria* show higher niche overlap when compared with other pairs (Figure 3), which suggests that speciation of the most recently diverged species pair is probably driven by geographic isolation (allopatry). Allopatry appears to be a common mode of speciation in alpine plants and birds in mountainous region (Drovetski et al., 2013; Schneeweiss, Winkler, & Schönswetter, 2017). Geographic isolation of recently diverged species with similar niche relationships has been observed in other herbaceous plants (Boucher, Zimmermann, & Conti, 2016). Since immigration has been identified as the major driver of Himalayan faunal diversity (Tamma, Marathe, & Ramakrishnan, 2016), the question of whether niche divergence occurred *insitu* or during immigration between the two recently diverged congeners remains unclear. Another close relative of these two pikas species is a trans‐Himalayan species, *O. curzoniae*. However, we recorded few detections of *O. curzoniae* in our study area and were unable to estimate the degree of niche overlap with its congeners. Since *O. curzoniae* occupied the highest elevations in our study area, they may be separated by elevation from their closest sister species, *O. nubrica* and *O. sikimaria* (Figure 2 and Supplementary Figure 2). In addition, it appears that the historical contact between *O. nubrica* and *O. curzoniae* may have contributed to similar mitochondrial DNA of these two species (Lissovsky, 2014; Lissovsky et al., 2019). Surprisingly, we did not encounter such signature of hybridization between *O. curzoniae* and *O. sikimaria*, despite a similar time of divergence.

The sister species *O. macrotis* and *O. roylii*, in *Conothao* subgenera, have a deeper divergence time (~4 myr) (Dahal et al., 2017) and exhibited niche divergence despite having comparatively higher geographic overlap (Figure 3 and Supplementary Table 5). The highest difference in their niche appears to be expressed by principal component 1 (Figure 3 and Supplementary Figure 3). The contributing variables to the two PCs of niche space are presented in the Supplementary Figure 3. A more localized study on the distribution of *O. roylii* and *O. macrotis* (Kawamichi, 1971) in a mountain range in the Khumbu region of Nepal suggest coexistence is achieved by elevational segregation. Our study captured the difference in climatic niches of pika species with the high geographic overlap, but the variables that promote coexistence perhaps needs a study at more localized scales. Whether the segregation is an expression of interspecific competition or the region is a contact zone of niche spaces of the two species remains to be investigated.

### 4.3 Niche breadth and geographic range size as proxies for vulnerability assessment to climate change

Pikas are highly responsive to changing climate conditions (Beever et al., 2010, 2011). Therefore, the current climatic niche breadth of pika species may provide insights into their adaptability to ongoing climate changes. A species' niche breadth informs us about the climatic tolerance and fitness optimum of a species across its distributional range (Cabrelli, Stow, & Hughes, 2014; Wright, 1932). Niche breadth is often seen to correlate with geographic range (Sheth & Angert, 2014)—species with large geographic ranges are expected to have a broader climatic tolerances and hence larger niche breadths (Slatyer, Hirst, & Sexton, 2013). Overall, among the four pikas in the Himalaya whose niche analyses were ascertained, we observed that the species with least geographic breadth has the smallest niche breadth. But, among the three species with wider geographic ranges, *O. nubrica, O. roylii,* and *O. macrotis,* we estimated highest niche breadth values for *O. nubrica* despite having smaller geographic breadth compared with *O. macrotis* and *O. roylii*. Using niche breadth and geographic range sizes as proxies for the adaptive potential of pikas to climate change, our study suggests that *O. sikimaria* perhaps is the most vulnerable species to future climate change assuming no niche evolution and no range expansion. Comparative habitat suitability models, forecasted to future climate change scenarios, could better refine the hypothesis of the vulnerability of *O. sikimaria* to future climate change.

*Ochotona roylii* has the least niche overlap (of approximately 0.3) with all species suggesting that it occupies the most distinct climatic niche space in the study area. On the other hand, the congener *O. macrotis* has highest niche overlap with all species under comparison except *O. roylii* (Figure 3). It is surprising that *O. roylii* has high niche breadth and still has lower overlap, and on the contrary, *O. macrotis* has smaller niche breadth but higher overlap with species under comparison.

## 5 IMPLICATIONS AND CAVEATS

This study is the first comprehensive effort to understand the distribution patterns of high‐altitude mammalian specialist species in a vulnerable and largely inaccessible mountain ecosystem. Our study is restricted to six pika species—a similar study including additional sister‐species pairs across the pika phylogeny would allow us to quantitatively test hypotheses related to niche evolution. Nevertheless, the current study shed light on previously unexplored aspects of distribution patterns of pikas by quantification of genetic, climatic niche, and geographic divergence. The hypothesis generated in this study should be tested in the entire distributional range of these species and across pika phylogeny. Future studies should consider including additional niche dimensions such as food resources and a fuller exploration of the role of competition among congeners. Though we documented few cases of local coexistence among species pairs, detailed sampling in areas of high pika species diversity (e.g., Annapurna, Ladakh and West Sikkim) is needed to better understand the role of biotic interactions.

## CONFLICT OF INTEREST

None declared.

## AUTHOR CONTRIBUTIONS

ND and UR designed the study. ND collected the samples, analyzed the data and wrote the manuscript. UR, SK, BN and RN helped in the analyses and corrected the manuscript. RPL helped in sampling.

## Data Availability

All sequences deposited to GenBank, accession numbers Arunachal Pradesh (MF614692—MF614719), North Sikkim (MN075978—MN076004), East Sikkim (MN076047—MN076109), South Sikkim (MN076110—MN076118), West Sikkim (MN076119—MN076137), Langtang (MN075969—MN075977, MN066155), Annapurna Conservation Area (MN076005—MN076046), Spiti (MN076138—MN076183), and Ladakh (MN075946—MN075968).
