# Supplementary material for: The role of geography, environment, and genetic divergence on the distribution of pikas in the Himalaya
Source: Ecol Evol. 2020 Jan 22;10(3):1539–51. doi: 10.1002/ece3.6007 (PMC7029102; doi:10.1002/ece3.6007)
Supplement: Supplementary file 1 [file ECE3-10-1539-s001.pdf]

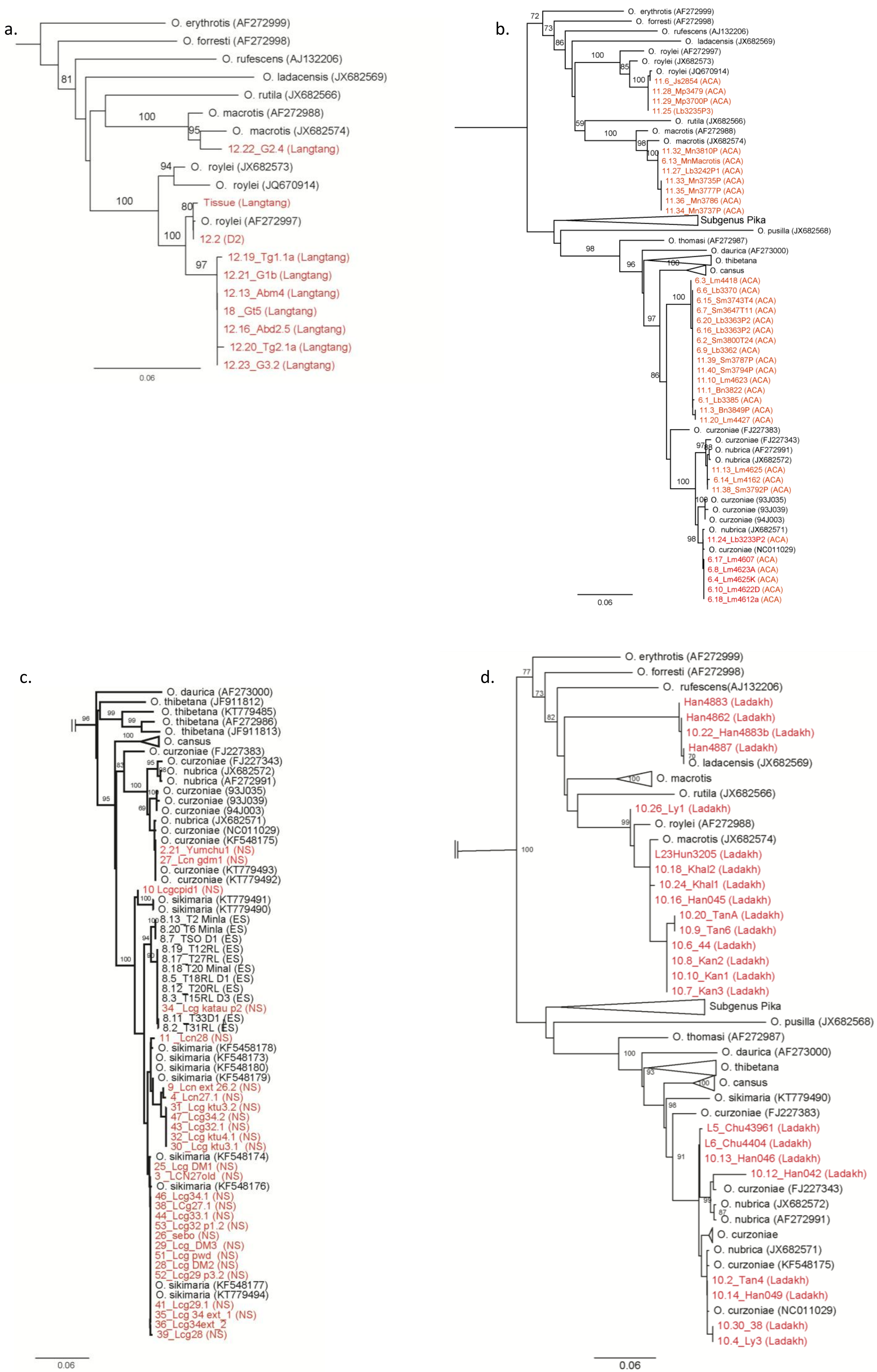

a.
O. erythrotis (AF272999)
O. forresti (AF272998)
O. rufescens (AJ132206)
O. ladacensis (JX682569)
O. rutila (JX682566)
O. macrotis (AF272988)
O. macrotis (JX682574)
12.22_G2.4 (Langtang)
O. roylei (JX682573)
O. roylei (JQ670914)
Tissue (Langtang)
O. roylei (AF272997)
12.2 (D2)
12.19_Tg1.1a (Langtang)
12.21_G1b (Langtang)
12.13_Abm4 (Langtang)
18 _Gt5 (Langtang)
12.16_Abd2.5 (Langtang)
12.20_Tg2.1a (Langtang)
12.23_G3.2 (Langtang)
81
100
95
94
100
80
100
97
0.06
b.
O. erythrotis (AF272999)
O. forresti (AF272998)
O. rufescens (AJ132206)
O. ladacensis (JX682569)
O. roylei (AF272997)
O. roylei (JX682573)
O. roylei (JQ670914)
11.6_Js2854 (ACA)
11.28_Mp3479 (ACA)
11.29_Mp3700P (ACA)
11.25 (Lb3235P3)
O. rutila (JX682566)
O. macrotis (JX682988)
O. macrotis (JX682574)
11.32_Mn3810P (ACA)
6.13_MnMacrotis (ACA)
11.27_Lb3242P1 (ACA)
11.33_Mn3735P (ACA)
11.35_Mn3777P (ACA)
11.36 _Mn3786 (ACA)
11.34_Mn3737P (ACA)
Subgenus Pika
O. pusilla (JX682568)
O. thomasi (AF272987)
O. daurica (AF273000)
O. thibetana
O. cansus
6.3_Lm4418 (ACA)
6.6_Lb3370 (ACA)
6.15_Sm3743T4 (ACA)
6.7_Sm3647T11 (ACA)
6.20_Lb3363P2 (ACA)
6.16_Lb3363P2 (ACA)
6.2_Sm3800T24 (ACA)
6.9_Lb3362 (ACA)
11.39_Sm3787P (ACA)
11.40_Sm3794P (ACA)
11.10_Lm4623 (ACA)
11.1_Bn3822 (ACA)
6.1_Lb3385 (ACA)
11.3_Bn3849P (ACA)
11.20_Lm4427 (ACA)
O. curzoniae (FJ227383)
O. curzoniae (FJ227343)
O. nubrica (AF272991)
O. nubrica (JX682572)
11.13_Lm4625 (ACA)
6.14_Lm4162 (ACA)
11.38_Sm3792P (ACA)
O. curzoniae (93J035)
O. curzoniae (93J039)
O. curzoniae (94J003)
O. nubrica (JX682571)
11.24_Lb3233P2 (ACA)
O. curzoniae (NC011029)
6.17_Lm4607 (ACA)
6.8_Lm4623A (ACA)
6.4_Lm4625K (ACA)
6.10_Lm4622D (ACA)
6.18_Lm4612a (ACA)
72
73
86
100
85
100
59
100
98
100
98
96
100
97
86
100
9788
100
100
98
0.06
c.
O. daurica (AF273000)
O. thibetana (JF911812)
O. thibetana (KT779485)
O. thibetana (AF272986)
O. thibetana (JF911813)
O. cansus
O. curzoniae (FJ227383)
O. curzoniae (FJ227343)
O. nubrica (JX682572)
O. nubrica (AF272991)
O. curzoniae (93J035)
O. curzoniae (93J039)
O. curzoniae (94J003)
O. nubrica (JX682571)
O. curzoniae (NC011029)
O. curzoniae (KF548175)
2.21_Yumchu1 (NS)
27_Lcn gdm1 (NS)
O. curzoniae (KT779493)
O. curzoniae (KT779492)
10 Lcgcpid1 (NS)
O. sikimaria (KT779491)
O. sikimaria (KT779490)
8.13_T2 Minla (ES)
8.20_T6 Minla (ES)
8.7_TSO D1 (ES)
8.19_T12RL (ES)
8.17_T27RL (ES)
8.18 T20 Minal (ES)
8.5_T18RL D1 (ES)
8.12_T20RL (ES)
8.3_T15RL D3 (ES)
34 _Lcg katau p2 (NS)
8.11_T33D1 (ES)
8.2_T31RL (ES)
11 _Lcn28 (NS)
O. sikimaria (KF5458178)
O. sikimaria (KF548173)
O. sikimaria (KF548180)
O. sikimaria (KF548179)
9_Lcn ext 26.2 (NS)
4_Lcn27.1 (NS)
31_Lcg ktu3.2 (NS)
47_Lcg34.2 (NS)
43_Lcg32.1 (NS)
32_Lcg ktu4.1 (NS)
30_Lcg ktu3.1 (NS)
O. sikimaria (KF548174)
25_Lcg DM1 (NS)
3_LCN27old (NS)
O. sikimaria (KF548176)
46_Lcg34.1 (NS)
38_LCg27.1 (NS)
44_Lcg33.1 (NS)
53_Lcg32 p1.2 (NS)
26_sebo (NS)
29_Lcg_DM3 (NS)
51_Lcg pwd (NS)
28_Lcg DM2 (NS)
52_Lcg29 p3.2 (NS)
O. sikimaria (KF548177)
O. sikimaria (KT779494)
41_Lcg29.1 (NS)
35_Lcg 34 ext_1 (NS)
36_Lcg34ext_2
39_Lcg28 (NS)
96
99
99
100
83
95
98
100
100
69
95
100
100
94
90
100
0.06
d.
O. erythrotis (AF272999)
O. forresti (AF272998)
O. rufescens(AJ132206)
Han4883 (Ladakh)
Han4862 (Ladakh)
10.22_Han4883b (Ladakh)
Han4887 (Ladakh)
O. ladacensis (JX682569)
O. macrotis
O. rutila (JX682566)
10.26_Ly1 (Ladakh)
O. roylei (AF272988)
O. macrotis (JX682574)
L23Hun3205 (Ladakh)
10.18_Khal2 (Ladakh)
10.24_Khal1 (Ladakh)
10.16_Han045 (Ladakh)
10.20_TanA (Ladakh)
10.9_Tan6 (Ladakh)
10.6_44 (Ladakh)
10.8_Kan2 (Ladakh)
10.10_Kan1 (Ladakh)
10.7_Kan3 (Ladakh)
Subgenus Pika
O. pusilla (JX682568)
O. thomasi (AF272987)
O. daurica (AF273000)
O. thibetana
O. cansus
O. sikimaria (KT779490)
O. curzoniae (FJ227383)
L5_Chu43961 (Ladakh)
L6_Chu4404 (Ladakh)
10.13_Han046 (Ladakh)
10.12_Han042 (Ladakh)
O. curzoniae (FJ227343)
O. nubrica (JX682572)
O. nubrica (AF272991)
O. curzoniae
O. nubrica (JX682571)
O. curzoniae (KF548175)
10.2_Tan4 (Ladakh)
10.14_Han049 (Ladakh)
O. curzoniae (NC011029)
10.30_38 (Ladakh)
10.4_Ly3 (Ladakh)
77
73
82
70
100
99
100
100
93
100
98
91
99
87
0.06

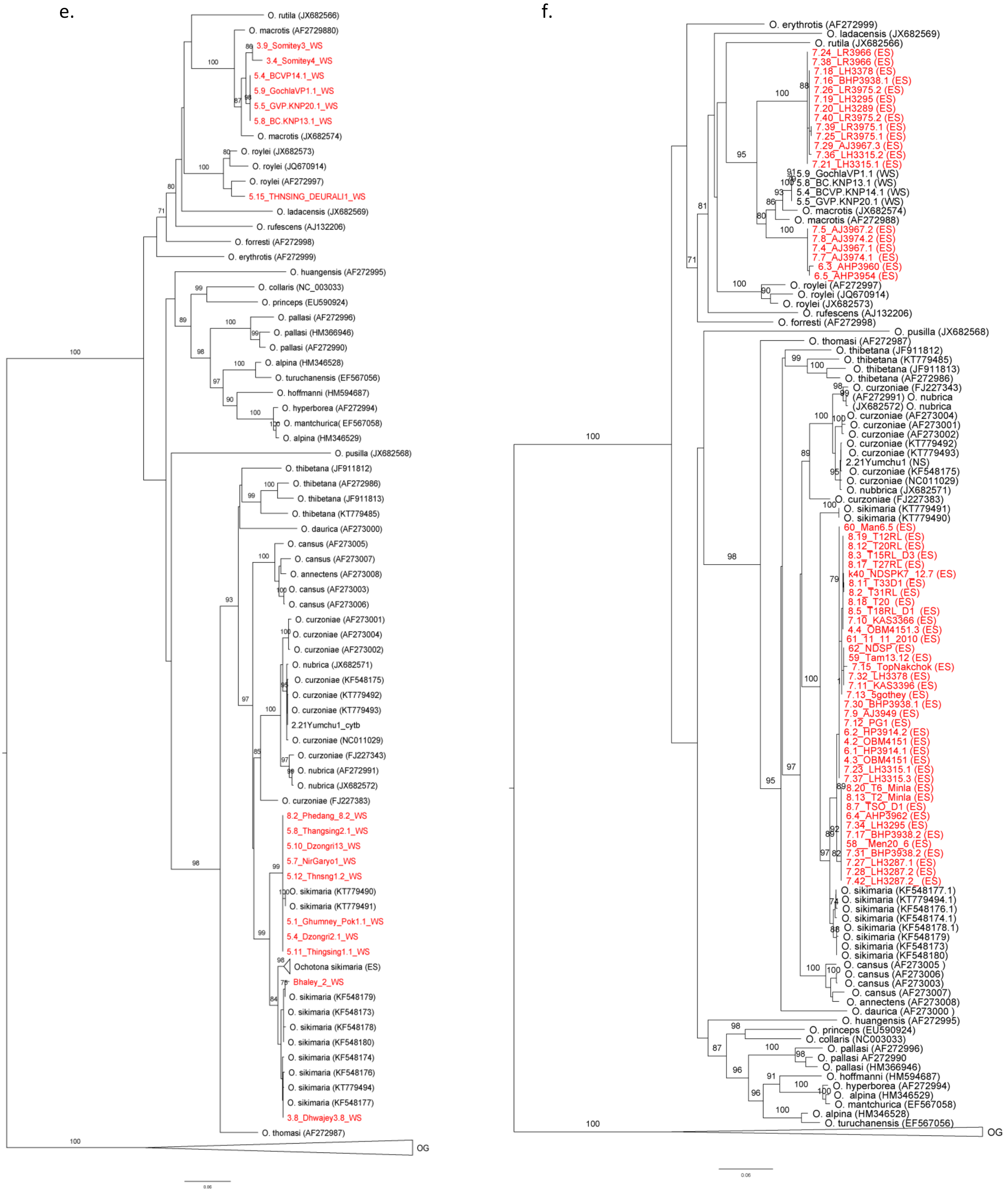

Supplementary figure 1 shows maximum likelihood tree constructed using RAxML GUI v1.3. The position of samples collected from (a) Langtang, (b) Annapurna Conservation Area (c) North Sikkim, (d) Ladakh, (e) West Sikkim, and (f) East Sikkim, are shown in red colour, and the comparison was made with valid species identified from Genbank. The scale bars in the bottom represent the mean number of nucleotide substitutions per site. The trees were constructed using partial cytochrome *b* sequences from pellets and tissue samples.

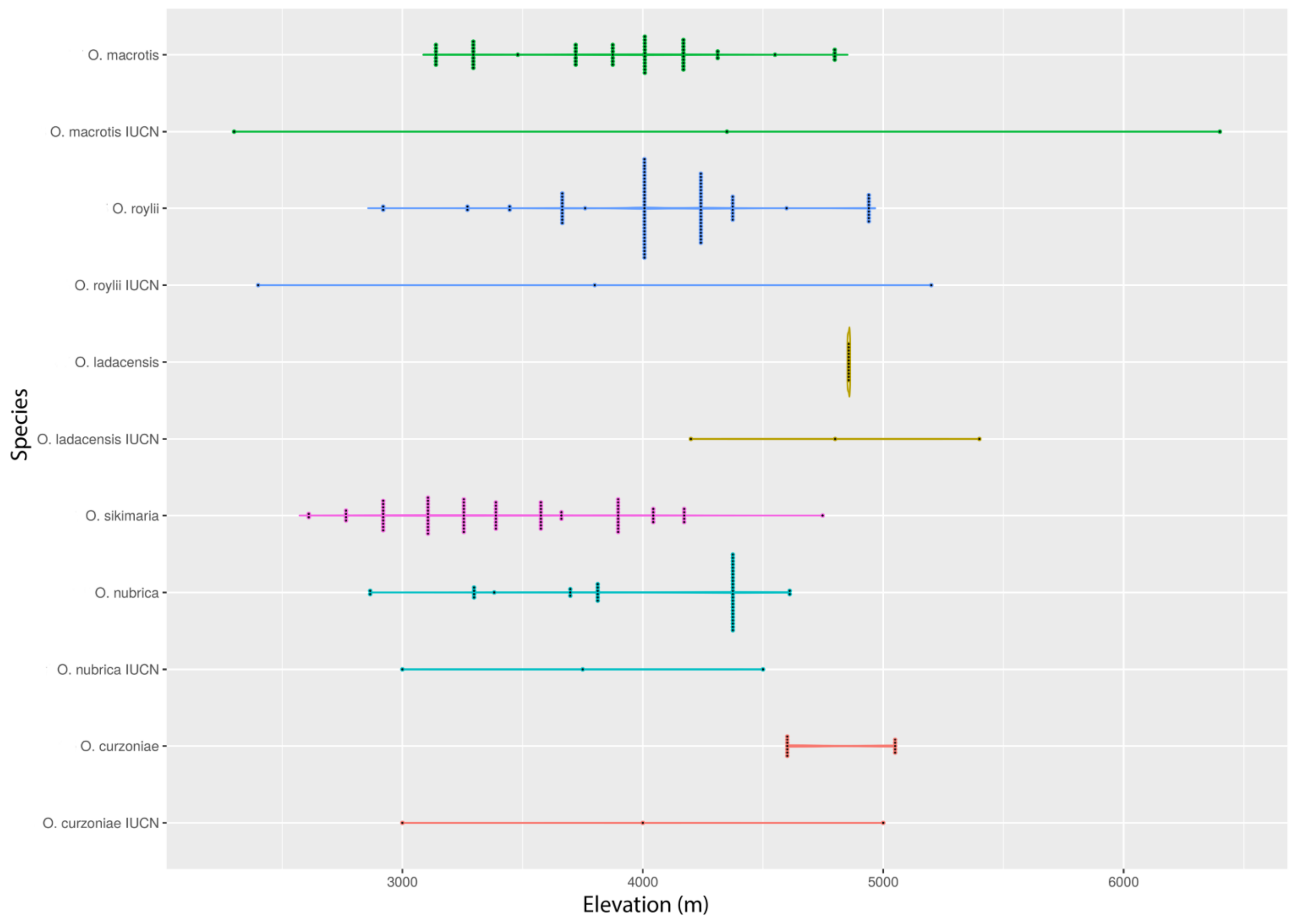

Supplementary figure 2: The elevational distribution of Himalayan pika species based on our observations and IUCN elevation range. The horizontal lines represent a species' elevation range, and the dots represent the number of samples at that elevation. The first three species belong to subgenus *Conothoa*, and the last three belong to subgenus *Ochotona*.

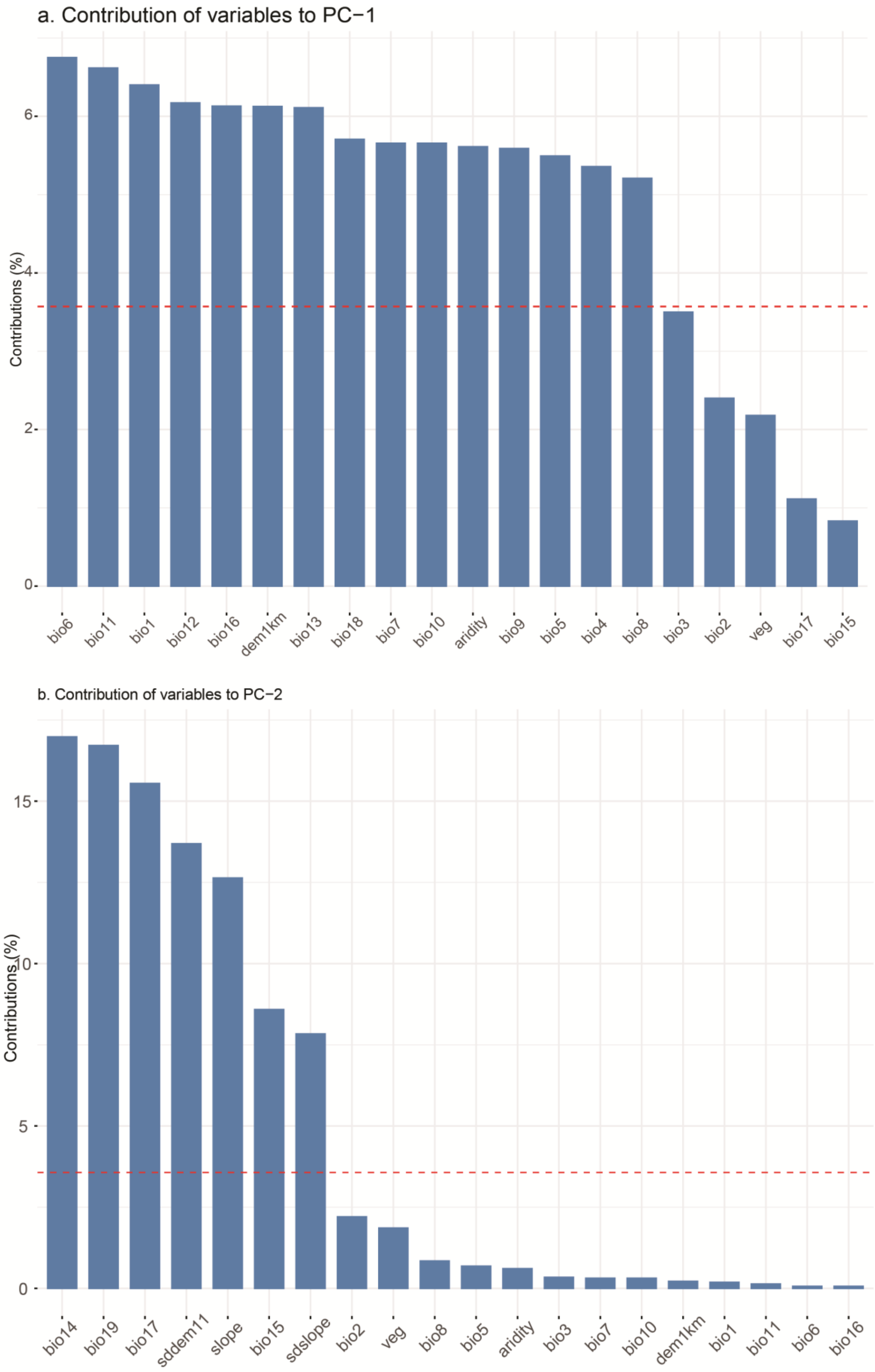

Supplementary figure 3: The bar plot shows the contribution of top 20 variables to PCA-env depicted in figure 3.

Supplementary table 1: Details of transects

Transect name, region	The elevation range of the region (in meters)	Month and year of sampling	Number of pellets	Number of tissue samples
Ladakh, Jammu & Kashmir	2841.5 - 4888	Aug – Sept. 2014	67	36
Spiti, Himachal Pradesh	3643 - 4997	Sept - Oct 2012 and 2013	87	17
Annapurna conservation area, Mustang, Nepal	2500 - 4625	Aug – sept 2013	59	21
Langtang National park, Rasuwa, Nepal	2015 - 4459	April 2012	24	1
Kyongnosla Alpine Sanctuary, Sikkim	3096- 4156.8	Nov 2010 and July 2013	95 (old & new)	35
Maenam Wildlife Sanctuary, Sikkim	2000- 3233.6	May 2011 and Sept. 2012	11	5
Kanchenjunga National Park, Sikkim	2160.1-4798.5	October 2011 and April 2013	34	9
Lachen, Sikkim	1987.2- 5067	May-June 2011	7	9
Lachung, Sikkim	2000 - 5067	April 2011	29	5
Barsey Rhododendron Sanctuary, Sikkim	2000- 3225.5	April 2011	nil	nil
Tawang, Arunachal Pradesh	3311.36-4347.6	Nov 2013 and September 2014	69	24

Supplementary table 2: Primer details.

Primer sequence	Annealing temperature	Amplicon type	Reference
L14724: CGAAGCTTGATATAAAAACCATCGTTG or GATATGAAAAACCATCGTTG	50	Mitochondria-cytochrome *b*	Irwin, et al., 1991
H15149: AACTGCAGCCCCTCAGAATGATATTTGTCCTCA or GCCCCTCAGAATGATATTTGTCCTCA	50	Mitochondria-cytochrome *b*	Irwin, et al., 1991
L14994A: GTCACCCATATTTGCCGAGATGTTAATTATGGC	Touchdown PCR described in section 2.2	Mitochondria-cytochrome *b*	unpublished, designed by Lissovsky (Moscow museum)
H15274A: AGGGTGGCTTTGTCTACTGAGAATCCG	Touchdown PCR described in section 2.2	Mitochondria-cytochrome *b*	unpublished, designed by Lissovsky (Moscow museum)

Supplementary table 3: Success rate of non-invasive species identification per region.

Location	Total pellets collected	Number of samples that worked	Number of samples that failed	% success
Ladakh	67	59	8	88.05
Spiti	87	71	16	81.6
Annapurna	59	45	14	76.27
Langtang	24	15	9	62.5
Sikkim	134	91	43	67.91
Arunachal Pradesh	69	31	38	44.92

Supplementary table 4: Details of environmental layers used for analyses.

Name	Code	Data type/ classification method	Source	Description
Elevation	DEM	continuous	Shuttle Radar Topography Mission (SRTM) https://lta.cr.usgs.gov/SRTM	30 m resolution layer rescaled to 1 km
Change in elevation	sdDEM	continuous	Derived from scaled DEM	Standard deviation of elevation difference in 2km neighbouring cells
Mean of elevation (meters)	meanDEM	continuous	Derived from scaled DEM	Mean within 2 km neighbouring cells
Vegetation	veg	Categorical data/IGBP classification scheme	MODIS Annual Land-Cover dataset (MCD12Q1) https://lpdaac.usgs.gov/dataset_discovery/modis/modis_products_table/mcd12q1	500 m resolution data resampled to 1 km resolution. Valid range 0-254.

Aridity Index	Aridity	continuous	Global Aridity Index (Global-Aridity) and Global Potential Evapo-Transpiration (Global-PET) http://www.csi.cgiar.org.	1 km resolution data. Annual average over the 1950-2000 period. Global-Aridity values need to be multiplied by 0.0001 to retrieve the values in the correct units. Aridity Index (AI) = MAP/ MAE, where: MAP = Mean Annual Precipitation and MAE = Mean Annual Potential Evapo-Transpiration
Slope	Slope	Continuous (in percentage)	Derived from scaled DEM	
Change in slope	Sdslope	Continuous (in percentage)	Derived from scaled DEM	Standard deviation of slope difference in 2km neighbouring cells
Aspect	East & north	Continuous	Derived from aspect	Eastness = sin (aspect) Northness = cos(aspect)
Bioclimatic layers 1 to 19	Bio 1 to bio 19	Continuous (different units)	Worldclim https://worldclim.org/version2approximately 1km resolution	Years 1970 to 2000

Supplementary table 5: Table showing niche overlap and geographic overlap between different pika species pairs. Bhattacharya affinity (BA) and Utilization distance overlap index (UDOI) represent non-directional geographic overlap indices. Probability of home range overlap (PHR), $PHR_{1,2}$ represents probability of species 2 being located in 1's geographic range and $PHR_{2,1}$ represents probability of species 1 being located in 2's geographic range.

**Species pairs**	**Niche Overlap (D)**	**Non-direction indices of Geographic overlap**		**Directional index of Geographic overlap**	
		BA	UDOI	$PHR_{1,2}$	$PHR_{2,1}$
O. nubrica /O. sikimaria	0.55	0.0	0.0	0.0	0.0
O. macrotis /O. sikimaria	0.64	0.25	0.09	0.09	0.97
O. macrotis /O. nubrica	0.53	0.16	0.03	0.11	0.27
O. macrotis/O. roylei	0.35	0.22	0.07	0.37	0.17
O. roylei /O. sikimaria	0.31	0.12	0.02	0.02	1
O. nubrica/O. roylei	0.32	0.25	0.08	0.21	0.43

Supplementary table 6: List of accession numbers generated for the study.

**Location**	**Accession numbers**
Arunachal Pradesh	MF614692 - MF614719
North Sikkim	MN075978 - MN076004
East Sikkim	MN076047 - MN076109
South Sikkim	MN076110 - MN076118
West Sikkim	MN076119 - MN076137
Langtang	MN075969 - MN075977, MN066155
Annapurna conservation area	MN076005 - MN076046
Spiti	MN076138 - MN076183
Ladakh	MN066156 - MN075968
